# Role of antibiotic use, plasma citrulline and blood microbiome in advanced non-small cell lung cancer patients treated with nivolumab

**DOI:** 10.1186/s40425-019-0658-1

**Published:** 2019-07-10

**Authors:** Julia Ouaknine Krief, Pierre Helly de Tauriers, Coraline Dumenil, Nathalie Neveux, Jennifer Dumoulin, Violaine Giraud, Sylvie Labrune, Julie Tisserand, Catherine Julie, Jean-François Emile, Thierry Chinet, Etienne Giroux Leprieur

**Affiliations:** 1 Department of Respiratory Diseases and Thoracic Oncology, APHP – Hopital Ambroise Pare, Boulogne-Billancourt, France; 2 EA 4340, UVSQ, Université Paris-Saclay, Boulogne-Billancourt, France; 3 Department of Biochemistry, APHP – Hopital Cochin, Paris, France; 4 Department of Pathology, APHP – Hopital Ambroise Pare, Boulogne-Billancourt, France

**Keywords:** Non-small cell lung cancer, Antibiotic, Plasma, Blood, Microbiome, Citrulline, Nivolumab, Biomarker

## Abstract

**Background:**

Recent data suggested a role of gut microbiota and antibiotic use on immune checkpoint inhibitors efficacy. We aimed to evaluate the impact of early use of antibiotic (EUA), blood microbiome and plasmatic citrulline (marker of the intestinal barrier) on nivolumab efficacy in non-small cell lung cancer (NSCLC).

**Methods:**

We included all consecutive patients with advanced NSCLC treated with nivolumab in our Department between 2014 and 2017. Blood microbiome was analyzed at month (M) M0 and M2. Citrulline rates were evaluated at M0, M2, M4 and M6.

**Results:**

Seventy-two patients were included (EUA in 42%). Overall survival (OS) was longer without EUA (median 13.4 months) than with EUA (5.1 months, *p* = 0.03). Thirty-five patients (49%) had plasma samples available. High citrulline rate (≥20 μM) at M0 was associated with tumor response (*p* = 0.084) and clinical benefit (nivolumab > 6 months) (*p* = 0.002). Median progression-free survival (PFS) was 7.9 months (high citrulline) vs 1.6 months (low citrulline) (*p* < 0.0001), and median OS were respectively non reached vs 2.2 months (*p* < 0.0001). Patients with EUA had lower median citrulline rates at M0: 21 μM (IQR 15.0–30.8) vs 32 μM (IQR 24.0–42.0) without EUA (*p* = 0.044). The presence of specific bacterial DNA in blood at M0 was associated with response and clinical benefit (Peptostreptococcae, Paludibaculum, Lewinella) or with tumor progression (Gemmatimonadaceae). Multivariate analyses on PFS and OS confirmed the prognostic role of citrulline and blood microbiome.

**Conclusions:**

EUA is associated with shorter OS with nivolumab and lower citrulline rates. Plasma citrulline and blood microbiome appear to be promising predictive factors of nivolumab efficacy.

**Electronic supplementary material:**

The online version of this article (10.1186/s40425-019-0658-1) contains supplementary material, which is available to authorized users.

## Background

Immune checkpoint inhibitors (ICIs), targeting programmed death-1 (PD-1) and its ligand programmed death ligand-1 (PDL1), are efficient drugs in advanced non–small-cell lung cancer (NSCLC) [1–4]. Nivolumab is an anti-PD1 antibody currently used in second-line or later therapy in advanced NSCLC, independently of PD-L1 expression [1, 2]. Primary resistance to ICIs in this setting remains common; therefore identifying new predictive biomarkers of response is urgently needed. Recent studies have suggested an impact of the gut microbiota in ICI efficacy [5–7]. Moreover, early use of antibiotics (EUA) (2 months before to 1 month after the beginning of ICI) decreases the efficacy of ICI [7–9]. Routy et al. showed that EUA was significantly associated with a shorter overall survival (OS) with ICI treatment (15.3 months without antibiotic versus 8.3 months, *p* < 0.01) [7]. This impact could be related to modifications of the gut microbiota by antibiotics [10–12], and bacterial translocation, interfering with anti-tumor immune response. Bacterial translocation can be explained by variations of both gut and systemic immune barriers [13]. Citrulline is an amino acid, produced almost exclusively by enterocytes of the small bowel mucosa [14]. Citrulline concentrations are independent from nutritional status. Citrulline rates are decreased in conditions causing a reduction of enterocyte mass like short bowel syndrome or villous atrophy diseases, and citrulline is a validated marker of the intestinal barrier, enterocytes function and leaky gut [15]. Blood microbiome, mainly but not exclusively constituted by gut bacteria, can be explored by 16S targeted metagenomic sequencing [16], and has been proven to contribute to the development of a variety of inflammatory diseases (atherosclerosis, cardiovascular disease, type II diabetes and neurodegenerative diseases) [17–19]. It has also been suspected that changes in blood microbiome might be associated with liver fibrosis in obese patients [20]. The role of blood microbiome and biomarker of intestinal barrier (like citrulline) remains unknown during ICI treatment. We aimed to study the impact of EUA, plasma citrulline and blood microbiome in patients with advanced NSCLC patients treated with nivolumab.

## Material and methods

### Patients

We included all consecutive patients with advanced non-small cell lung cancer treated with nivolumab (3 mg/kg d1d15, given intravenously) as second or later line therapy in the Department of Respiratory Diseases and Thoracic Oncology of the Hospital Ambroise Pare, Boulogne-Billancourt, France.

Demographic and pathological data were collected. Patient records were reviewed to determinate if they had been treated with oral or intravenous antibiotherapy two months before until one month after the beginning of nivolumab, defining EUA, given for any reason. The class of antibiotic, indication, route of administration and duration were collected. Tumor response was determined by Response Evaluation Criteria in Solid Tumors (RECIST) version 1.1, based on CT scans every 4 injections, by an expert thoracic radiologist and validated in multi-disciplinary meeting. Overall response rate (ORR) was defined as the proportion of patients with complete or partial response. Clinical benefit was defined as nivolumab given more than 6 months.

### Citrulline and blood microbiome

Plasma citrulline concentration was prospectively measured at months (M) M0, M2, M4 and M6 by ion exchange chromatography. Low citrulline rate was defined as concentration less than 20 μM, and high citrulline rate as concentration equal or more than 20 μM.

Blood microbiome was assessed by sequencing of variable regions (V3-V4) of the 16S rRNA bacterial gene at M0 (for correlation with citrulline, tumor response and clinical benefit) and M2 (for correlation with EUA), (Vaiomer, Toulouse, France). Briefly, PCR amplification was performed using 16S universal primers targeting the V3-V4 region of the bacterial 16S ribosomal gene. The joint pair length was set to encompass 467 base pairs amplicon thanks to 2 × 300 paired-end MiSeq kit (V3). For each sample, a sequencing library was generated by addition of sequencing adapters. The detection of the sequencing fragments was performed using MiSeq Illumina® technology. The targeted metagenomic sequences from microbiota were analyzed using the bioinformatics pipeline established by Vaiomer from the FROGS guidelines. Operational taxonomic units (OTUs) were produced with single-linkage clustering and taxonomic assignment was performed in order to determine community profiles [21].

### PDL1 immunohistochemistry

PDL1 immunohistochemistry (IHC) was performed using an automated method (Leica) and the E1L3N anti-PD-L1 antibody (Cell Signalling Technology) diluted to the 1/80th on 4 μm-slides from the treatment-naïve diagnostic samples. The assay was performed using human amygdala as positive control, and IgG as isotype negative control. The IHC was considered as being positive if at least one tumour cell out of 100 analyzed tumor cells was positively stained.

### Statistical analyses

We compared continuous variables using Mann-Whitney test or Fisher test and categorical variables using Chi^2^ test, according to EUA and citrulline rate (low versus high). ORR and clinical benefit rates comparisons were also performed by Chi^2^ test, according to EUA and citrulline rate (low versus high). Concerning blood microbiome analyses, reads obtained from the MiSeq sequencing system have been processed using Vaiomer bioinformatics pipeline. The steps included quality-filtering, clustering into OTUs with the Swarm algorithm and taxonomic affiliation. Linear discriminant analysis (LDA) Effect Size (LEfSe) algorithm was used to identity statistically significant differences in microbiome composition according to clinical condition (tumor response, clinical benefit, citrulline rates, EUA) [22]. OS and progression-free survival (PFS) analyses were performed using Kaplan–Meier method (*p*-value calculated by log rank test). Multivariate analyses on OS and PFS were performed using Cox proportional hazards model. Statistical analyses (except blood microbiome analyses) were performed using Xlstat 2018 (Addinsoft, France).

## Results

### Patients’ characteristics

Seventy-two patients were treated with nivolumab between July 2014 and September 2017. Median duration of nivolumab treatment was 87 days (interquartile range IQR 36–138). Median number of nivolumab injections was 6 (IQR IQR 3–9). At the end of the follow-up, 11 patients were still receiving nivolumab. Mean follow-up time was 500 days (IQR 401–599).

Patients were mostly male (62%), median age 68.8 year-old (IQR 62.7–73.7), former or current smokers (87%), PS 0–1 at the beginning of nivolumab (63%), with stage IV disease (86%) (Table 1). 99% of the patients were Caucasian (only one Asian patient was noted in the group who did not receive EUA). Forty-five patients (63%) had an adenocarcinoma and 62 (86%) received 1 line of treatment before nivolumab.

**Table 1:** Characteristics of the patients, according to early use of antibiotics (EUA) and citrulline rates at baseline

	All (*n* = 72)	No EUA (*n* = 42)	EUA (*n* = 30)	*p*-value	High citrulline (*n* = 25)	Low citrulline (*n* = 10)	*p*-value
Age (median, IQR)	68.8 (62.7–73.7)	69.0 (63.4–73.4)	67.8 (58.4–73.7)	0.479	69.3 (63.5–72.7)	63.6 (56.5–67.1)	0.07
Sex							
male	45 (62)	26 (62%)	19 (63%)	0.902	16 (64%)	5 (50%)	0.445
female	27 (38)	16 (38%)	11 (37%)		9 (36%)	5 (50%)	
Smoking status							
current	29 (40)	20 (48%)	9 (30%)	0.062	11 (44%)	3 (30%)	0.534
former	34 (47)	15 (35%)	19 (63%)		12 (48%)	5 (50%)	
never	9 (13)	7 (17%)	2 (7%)		2 (8%)	2 (20%)	
Histological type							
adenocarcinoma	45 (63)	27 (64%)	18 (60%)	0.592	19 (76%)	4 (40%)	0.019
squamous carcinoma	14 (19)	9 (22%)	5 (17%)		5 (20%)	2 (20%)	
other	13 (18)	6 (14%)	7 (23%)		1 (4%)	4 (40%)	
Mutational status							
KRAS	26 (36)	12 (29%)	14 (47%)	0.292	10 (40%)	3 (30%)	0.394
EGFR	2 (3)	1 (2%)	1 (3%)		1 (4%)	1 (10%)	
BRAF	3 (4)	1 (2%)	2 (7%)		1 (4%)	2 (20%)	
None or rare mutation	41 (57)	28 (67%)	13 (43%)		13 (52%)	4 (40%)	
Albumin				0.889			0.104
< 30 g/l	12 (17)	7 (17)	5 (17)		2 (8)	3 (30)	
≥ 30 g/l	57 (79)	32 (76)	25 (83)		22 (88)	7 (70)	
unknown	3 (4)	3 (7)	0 (0)		1 (4)	0 (0)	
Hemoglobin (median, IQR)	11.6 (10.0–12.7)	12.1 (11.3–13.0	10.5 (9.5–11.8)	0.006	12.3 (11.3–13.4)	9.9 (8.7–11.2)	0.006
Metastatic sites							
brain	8 (11%)	5 (12%)	3 (10%)	0.800	6 (24%)	0	0.089
liver	10 (14%)	5 (12%)	5 (17%)	0.565	2 (8%)	2 (20%)	0.313
≥ 3 sites	16 (22%)	7 (17%)	9 (30%)	0.180	3 (12%)	2 (20%)	0.541
Stage							
IIIB	10 (14)	5 (12%)	1 (3%)	0.195	3 (12%)	2 (20%)	0.541
IV	62 (86)	37 (88%)	29 (97%)		22 (88%)	8 (80%)	
Performance status							
0–1	45/71 (63)	27/41 (66%)	18 (60%)	0.216	22 (88%)	3 (30%)	0.001
2	26/71 (37)	14/41 (34%)	12 (40%)		3 (12%)	7 (70%)	
Number of lines before nivolumab							
1	62 (86%)	37 (88%)	26 (87%)	0.587	22 (88%)	9 (90%)	0.867
> 2	10 (14)	5 (12%)	4 (13%)		3 (12%)	1 (10%)	
PD-L1 IHC							
< 1%	8 (11)	6 (14)	2 (7)	0.072	6 (24)	2 (20)	0.900
≥ 1%	15 (21)	5 (12)	10 (33)		11 (44)	4 (40)	
unknown	49 (68)	31 (74)	18 (60)		8 (32)	4 (40)	

*EUA* early use of antibiotics. Variables expressed as n (%) unless otherwise specified. High citrulline: ≥20 μM; low citrulline: < 20 μM. *P*-values calculated by Mann-Whitney test or Chi^2^ test

PD-L1 status was available for 23 patients (32%). From these patients, 15 patients (65%) had positive (≥1%) PD-L1 staining, and 10 patients (43%) had high PD-L1 expression (≥50%).

ORR with nivolumab was 29% (*n* = 21) and clinical benefit rate was 28% (*n* = 20). Median PFS was 2.9 months (IQR 1.8–4.8) and median OS was 11.1 months (IQR 5.6-NR).

### EUA

**Fig. 1:**
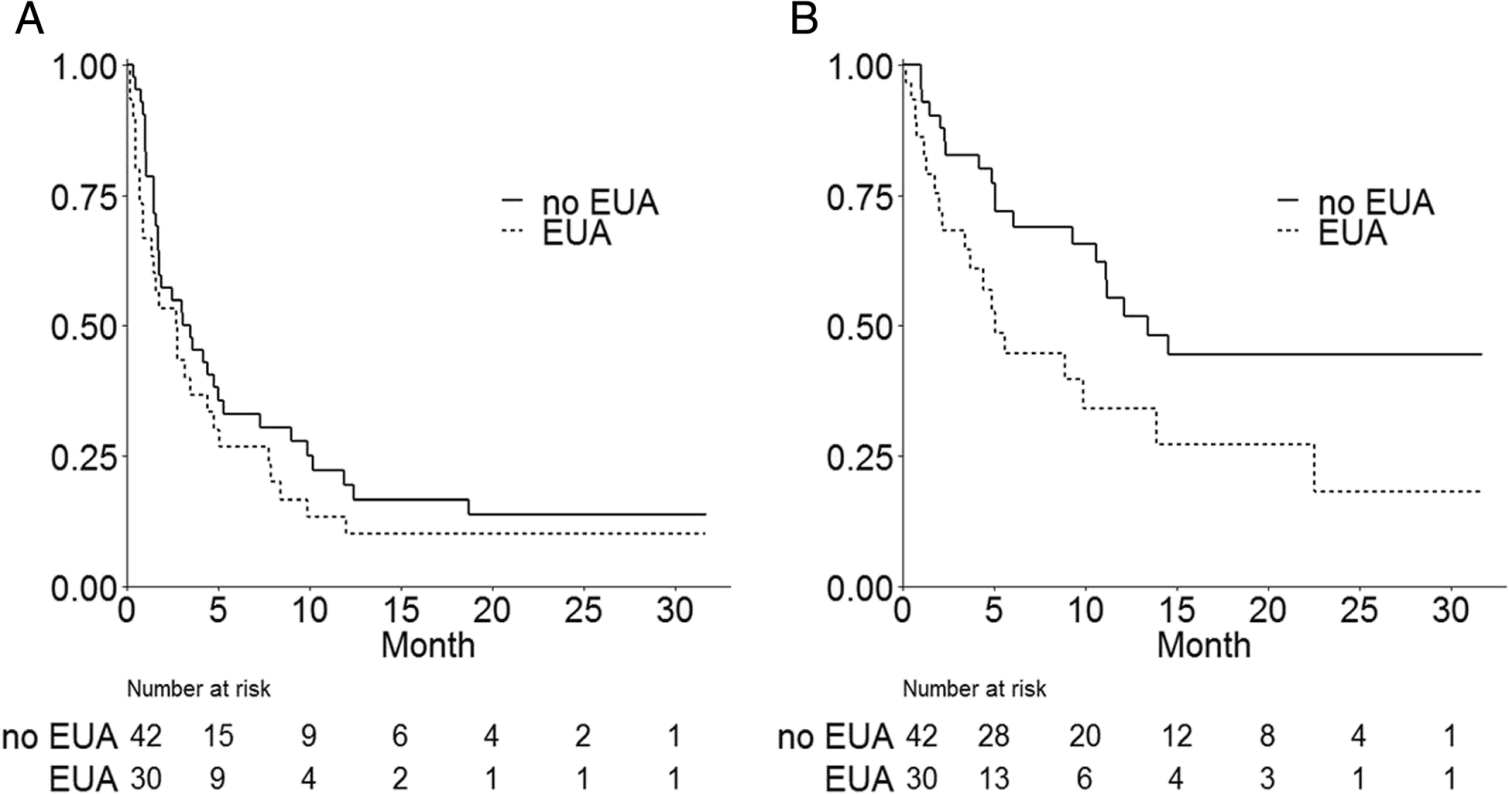
Progression-free survival (**a**) and overall survival (**b**) according to early use of antibiotics (EUA)

Thirty patients (42%) received EUA. Median duration of antibiotic treatment was 9.5 days (IQR 7–14). No significant clinical or histomolecular difference was observed between patients with or without EUA, except lower hemoglobin rate in case of EUA **(**Table 1). EUA was prescribed 51 times on the 30 patients (one patient could receive EUA several times). The most frequently prescribed antibiotics were beta-lactamines (*n* = 36/51) and vancomycine (*n* = 4/51) **(**Additional file 1: Figure S1**)**. Oral administration was predominant (65%) and most common indications were respiratory infections (*n* = 22 on the 45 available indications), intestinal infections (*n* = 5/45), skin infections (*n* = 4/45) and catheter infections (*n* = 4/45) **(**Additional file 1: Figure S2**)**. Thirty-three percent of the patients who received EUA were hospitalized (*n* = 10/30). ORR was 37% (*n* = 11) with EUA vs 24% (*n* = 10) without EUA (*p* = 0.276). Clinical benefit rate was 27% (*n* = 8) with EUA vs 29% (*n* = 12) without EUA (*p* = 0.859). Median PFS with and without EUA were respectively 2.8 months (IQR 1.4–5.1) and 3.3 months (IQR 1.8–7.3) (*p* = 0.249) (Fig. 1a). Median OS were respectively 5.1 months (IQR 3.4-not reached NR) and 13.4 months (IQR 10.6-NR) (*p* = 0.027) (Fig. 1b). Considering the use of antibiotics only during the month before nivolumab, median PFS was 1.8 months with antibiotics vs 3.0 months without (*p* = 0.341) and OS were 5.1 vs 13.3 months (*p* = 0.039), respectively.

### Plasma citrulline

**Fig. 2:**
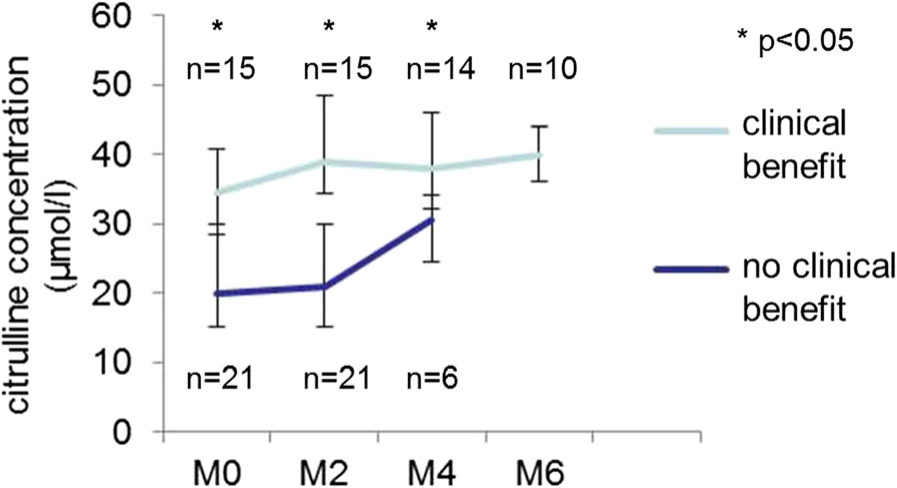
Evolution of plasma citrulline concentrations during nivolumab treatment, according to clinical benefit

Thirty-five patients accepted plasma collection for citrulline and blood microbiome analyses. Ten patients (29%) had low citrulline rates at baseline (< 20 μM). These patients had more often PS 2 (70%) than patients with high citrulline rates (12%) (*p* = 0.001), and lower hemoglobin rate (*p* = 0.006) (Table 1). Baseline citrulline tended be associated with ORR (52% with high citrulline vs 20% with low citrulline, *p* = 0.084) and was associated with clinical benefit (56% with high citrulline vs 0% with low citrulline, *p* = 0.002). Patients with clinical benefit of nivolumab had significantly higher plasmatic citrulline rates than other patients, at M0, M2 and M4, keeping high rates at M6 (Fig. 2).

**Fig. 3:**
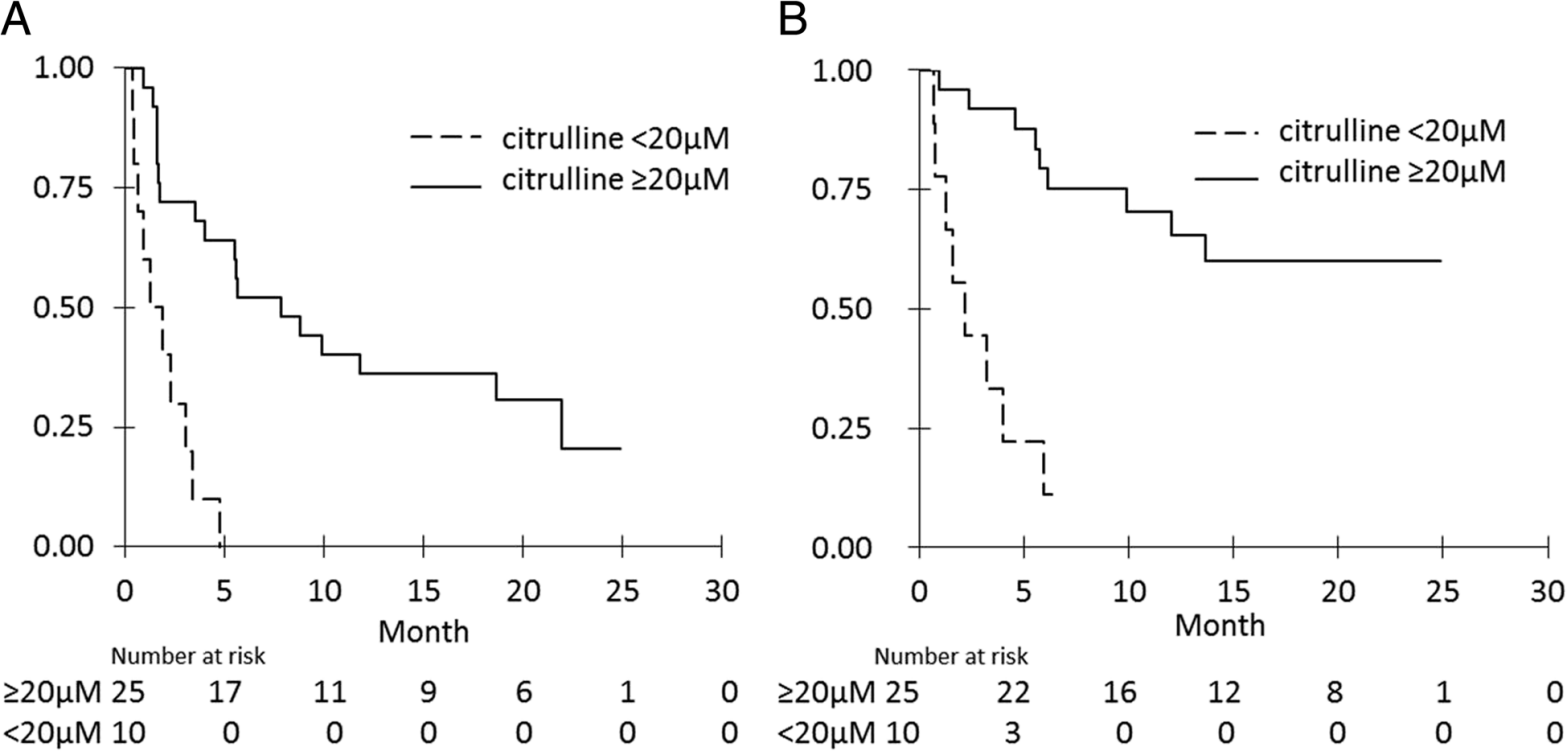
Progression-free survival (**a**) and overall survival (**b**) according to citrulline rates (high vs low)

Patients with high citrulline rates at M0 had significant higher PFS and OS. Median PFS was 7.9 months with high citrulline at M0 vs 1.6 months with low citrulline (*p* < 0.0001) (Fig. 3a) and median OS were respectively not reached and 2.2 months (*p* < 0.0001) (Fig. 3b).

Citrulline rates at M0 were available for 18 patients with EUA and 17 without EUA. Patients who received antibiotics in the 2 months before the beginning of nivolumab had lower median citrulline concentrations: 21 μM (IQR 15.0–30.8) vs 32 μM (IQR 24.0–42.0) (*p* = 0.044). Citrulline rates were independent of PDL1 expression in immunohistochemistry: 11 patients with high citrulline rate at M0 (44%) had positive PD-L1 staining, versus 4 patients with low citrulline rate (40%) (*p* = 0.900).

### Blood microbiome

Blood microbiome analyses were feasible for all 35 patients at M0. Most bacteria were from the Proteobacteria phylum (39%), followed by Bacteroidetes (30%), Actinobacteria (20%) and Firmicutes (7%) **(**Additional file 1: Figure S3**)**. This repartition was as expected in the literature [16].

**Fig. 4:**
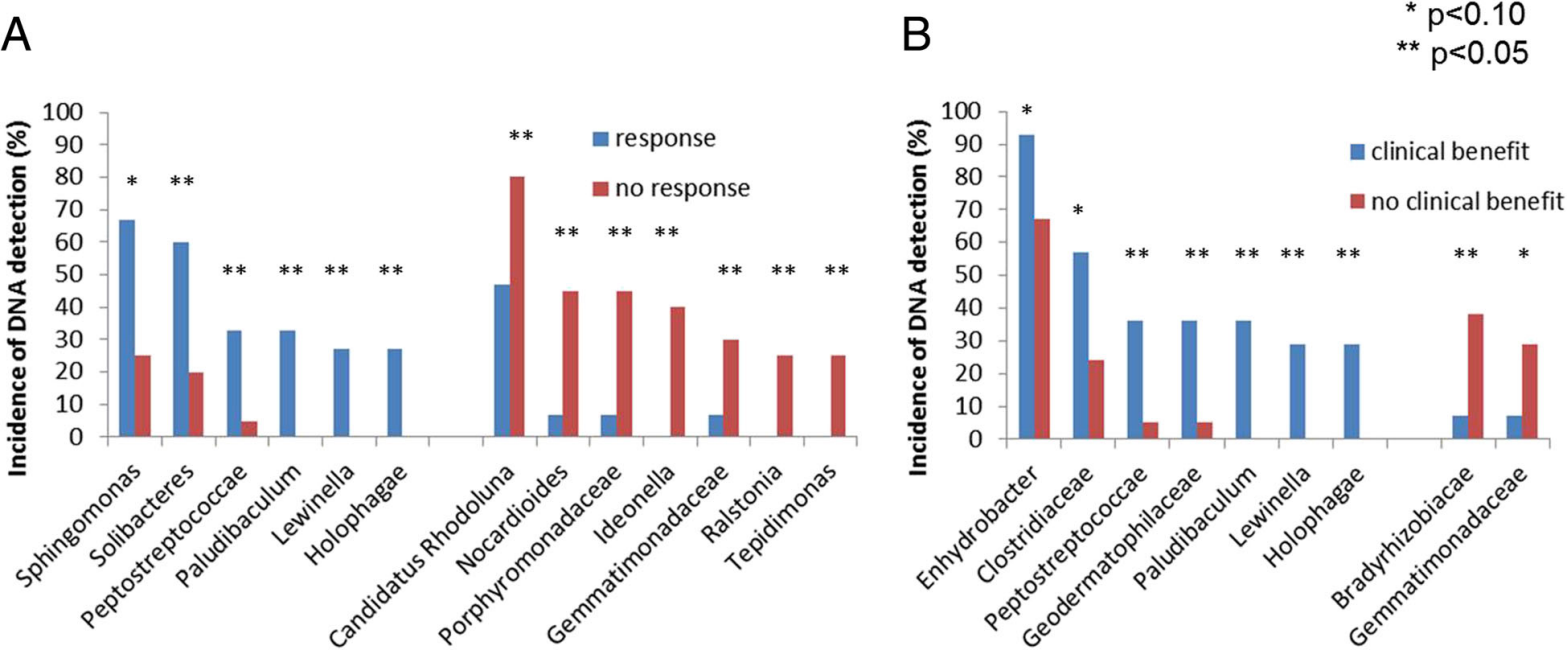
Incidence of DNA detection in blood at baseline of bacteria associated with tumor response (**a**) and clinical benefit (**b**)

Patients experiencing response and/or clinical benefit had different blood microbiome profile at M0 **(**Fig. 4, Additional file 1: Figure S4**,** Additional file 1: Figure S5**)**. The detection of Peptostreptococcaceae (*n* = 6), Lewinella (*n* = 4), Paludibaculum (*n* = 5) and Holophagae (*n* = 4) at M0 was significantly associated with both clinical benefit and response. Blood microbiome of patients who did not experience clinical benefit and/or tumor response was significantly enriched in Gemmatimonadaceae at M0 (*n* = 7). The presence of Gemmatimonadaceae DNA in blood at M0 was associated with both low response rate (14% vs 50% for other patients, *p* = 0.09) and high rate of progression at M2 (86% vs 29% for other patients, *p* = 0.006).

No difference in term of relative microbiome composition was observed according to citrulline levels. However, at M2, in patients who did not receive EUA, microbiome profile was enriched in Solibacteres which are associated with tumor response, compared to patients who received EUA **(**Additional file 1: Figure S6).

### PFS and OS multivariate analyses

Multivariate analysis on PFS including EUA (yes vs no), PS (0–1 vs 2), hemoglobin rate (continuous variable), albumin rate (< 30 g/l vs ≥30 g/l), Kras mutation (yes vs no), PDL1 status (< 1 vs ≥1%), citrulline concentration at baseline (< 20 μm vs ≥20 μM) and presence of Gemmatimonadaceae on blood microbiome analysis at baseline was performed. Low citrulline rate (hazard ratio HR = 3.8; IC95% 1.4–99.9; *p* = 0.008), PS 2 (HR = 2.1; IC95% 1.1–4.1; *p* = 0.023), and hemoglobin rate (HR = 0.8; IC95% 0.0–0.9; *p* = 0.005) were independently associated with PFS (Table 2). The presence of Gemmatimonadaceae on blood microbiome analysis at baseline tended to be associated with worse PFS (HR = 2.9, IC95% 0.9–9.2; *p* = 0.073).

**Table 2:** Multivariate analyses (Cox model) on PFS and OS

	Progression-free survival		Overall survival	
	HR (IC95%)	*p*-value	HR (IC95%)	*p*-value
EUA	1.6 (0.6–2.2)	0.645	2.2 (1.1–4.8)	0.038
PS 2	2.1 (1.1–4.1)	0.023	1.8 (0.7–4.4)	0.198
Hg rate (continuous)	0.8 (0.0–0.9)	0.005	0.8 (0.6–1.1)	0.174
Alb rate < 30 g/dl	1.1 (0.5–2.3)	0.908	0.5 (0.2–1.5)	0.216
Kras mutation	0.7 (0.4–1.2)	0.181	0.4 (0.0–0.9)	0.020
PDL1 ≥ 1%	1.6 (0.6–4.2)	0.295	3.0 (0.9–10.8)	0.085
Citrulline < 20 μM	3.8 (1.4–99.9)	0.008	2.4 (0.6–10)	0.222
Presence of Gemmatimonadaceae	2.9 (0.9–9.2)	0.073	16.4 (3.9–68.5)	< 0.001

*HR* hazard ratio, *EUA* early use of antibiotics, *PS* Performance status, *Hg* hemoglobin, *Alb* albumin

Multivariate analysis on OS including the same variables showed that EUA (HR = 2.2; IC95% 1.1–4.8; *p* = 0.038), Kras mutation (HR = 0.4; IC95% 0.0–0.9; *p* = 0.020) and presence of Gemmatimonadaceae on blood microbiome analysis at baseline (HR = 16.4; IC95% 3.9–68.5; *p* < 0.001) were independently associated with worse OS (Table 2).

## Discussion

In our study, EUA was associated with shorter OS with nivolumab, a different blood microbiome profile and lower rates of citrulline. High citrulline rate was associated with longer PFS and OS, a better response and clinical benefit to nivolumab. Blood microbiome analyses showed that several bacterial families were associated with response and/or clinical benefit, and that EUA impacted blood mircobiome composition.

Concerning the impact of EUA on ICI efficiency, our results correlated with several published studies [7–9], with poor survival associated with EUA. Routy et al. showed in a cohort of 249 patients diagnosed with advanced NSCLC (*n* = 140), renal cell carcinoma (*n* = 67) and urothelial cancer (*n* = 42), treated with ICIs, that EUA was associated with decreased PFS from 4.1 to 3.5 months (*p* = 0.017) and OS from 20.6 to 11.5 months (*p* < 0.01) [7]. Another recent study on a cohort of NSCLC antibiotic-treated patients (*n* = 239), PFS and OS were also significantly shorter with antibiotics (median PFS: 1.9 vs 3.8 months, p = 0.03; median OS: 7.9 vs 24.6 months, *p* < 0.01) [8]. In our study, OS was significantly decreased with EUA while PFS was not. A lack of power may explain the absence of significant difference on PFS.

Antibiotics are able to alter gut microbiota composition [23]. Among blood microbiome of patients who did not receive EUA, we highlighted the higher incidence of Solibacteres at M2, associated with tumor response, compared to microbiome profile of patients who received EUA. Moreover, we found that the presence of particular bacteria families at M0 was associated with outcome with nivolumab. Compared to Routy’s results [7], we confirmed that Firmicutes family, which Peptostreptococcaceae belong to, is associated with response to ICI. However, we found that several other bacteria families were associated with nivolumab outcomes, although they were not described in other published studies. This difference could be explained by the fact that we analyzed blood microbiome and not gut microbiota. It has been shown that the gut barrier plays a role of filter, limiting the translocation of specific microbiota to blood. Unlike other studies that analyzed gut microbiota in patients treated with ICI, we chose to study blood microbiome. Blood microbiome has already been evaluated in other diseases [16–20], but not in oncology yet. As far as we know, our work is the first to describe the blood microbiome profile in patients with cancer and to show the impact of blood microbiome on ICI efficacy. Blood microbiome offers the possibility to be easily collected, without any technical restriction or physical impairments that could interfere with collection of stools for gut microbiota analyses.

Moreover, our study is the first to our knowledge to propose citrulline as a predictive marker of nivolumab efficacy. In western countries, 97.5% of healthy subjects with normal intestinal and renal functions have citrulline blood concentration higher than 20 μM [15]. Inflammation and albumin levels do not interfere with plasma citrulline concentrations [15]. Considering citrulline as a validated marker of enterocyte function, we showed that EUA decreases plasma citrulline rates and that low concentrations were associated with absence of clinical benefit and poor survival with nivolumab. This might imply that citrulline is not only a marker of enterocyte function but reflects also gut dysbiosis induced by antibiotics, influencing tumor immunomodulation. In our study, patients with low basal citrulline rate had more often an altered PS (PS 2) compared to other patients. However, the negative role of PS 2 on nivolumab outcome still remains an unanswered question, as these patients were often excluded from clinical trials. Several retrospective studies suggested a negative impact of PS 2 on outcome with ICIs [24, 25]. We confirmed this association in multivariate analyses on PFS. Importantly, we also showed in multivariate analysis that low citrulline rate at M0 was associated with poor PFS with nivolumab (HR = 3.8; IC95% 1.4–99.9), independently from PS and nutritional status.

Our study has several limits. This is a monocentric retrospective study. The number of patients is small, limiting the power of statistical analyses. Moreover, no stool samples were collected in our study for correlation between gut and blood microbiome. However, as discussed earlier, we expect that blood microbiome differs from gut microbiota composition, due to the filter role of intestinal barrier. This study has also several strengths. All blood samples were collected prospectively. We were able to perform sequential citrulline and blood microbiome assessments during nivolumab treatment. We were also able to correlate EUA, intestinal barrier (reflected by citrulline rate) and blood microbiome. At last, we demonstrated the feasibility and impact of blood microbiome analysis in patients treated with ICIs. Our multivariate analyses confirmed the independent role of citrulline rate and blood microbiome profile on survival with nivolumab.

## Conclusions

In conclusion, we confirmed that nivolumab efficacy is impacted by external factors that modify the intestinal barrier and microbiota, as EUA. Plasma citrulline and blood microbiome appear to be promising predictive factors in this situation. Further prospective studies with larger number of patients and other ICI treatment strategies are needed to validate these results.

## Additional file

Additional file 1: **Figure S1.** Repartition of the antibiotic classes in early use of antibiotics (EUA) group for each prescription (*n* = 51). **Figure S2.** Therapeutic indications of EUA (*n* = 51). **Figure S3.** Main bacteria phylum in blood at baseline in the overall population (*n* = 35). **Figure S4.** LEFSE diagram of blood microbiome at M0 according to tumor response. **Figure S5.** LEFSE diagram of blood microbiome at M0 according to clinical benefit **Figure S6.** LEFSE diagram of blood microbiome at M2 according to early use of antibiotic (EUA). (DOCX 2147 kb)

## Data Availability

The datasets used and/or analyzed during the current study are available from the corresponding author on reasonable request.

## Abbreviations

Alb: Albumin
EUA: Early use of antibiotics
Hg: Hemoglobin
ICI: Immune checkpoint inhibitor
IHC: Immunohistochemistry
IQR: Interquartile range
LDA: Linear discriminant analysis
LEFSe: LDA Effect Size
M: Month
NSCLC: Non-small cell lung cancer
ORR: Overall response rate
OS: Overall survival
OTU: Pperational taxonomic unit
PD1: Programmed death-1
PDL1: Programmed death ligand-1
PFS: Progression-free survival
RECIST: Response Evaluation Criteria in Solid Tumors
