# Supplementary material for: Role of antibiotic use, plasma citrulline and blood microbiome in advanced non-small cell lung cancer patients treated with nivolumab
Source: J Immunother Cancer. 2019 Jul 10;7:176. doi: 10.1186/s40425-019-0658-1 (PMC6621972; doi:10.1186/s40425-019-0658-1)
Supplement: Supplementary file 1 — Figure S1. Repartition of the antibiotic classes in early use of antibiotics (EUA) group for each prescription (n = 51). Figure S2. Therapeutic indications of EUA (n = 51). Figure S3. Main bacteria phylum in blood at baseline in the overall population (n = 35). Figure S4. LEFSE diagram of blood microbiome at M0 according to tumor response. Figure S5. LEFSE diagram of blood microbiome at M0 according to clinical benefit Figure S6. LEFSE diagram of blood microbiome at M2 according to early use of antibiotic (EUA). (DOCX 2147 kb) [file 40425_2019_658_MOESM1_ESM.docx]

**Additional files**

**ADDITIONAL FIGURE 1:** Repartition of the antibiotic classes in early use of antibiotics (EUA) group for each prescription (n=51). PO: per os. IV: intra-venous. C3G: cephalosporin, 3^rd^ generation.

**
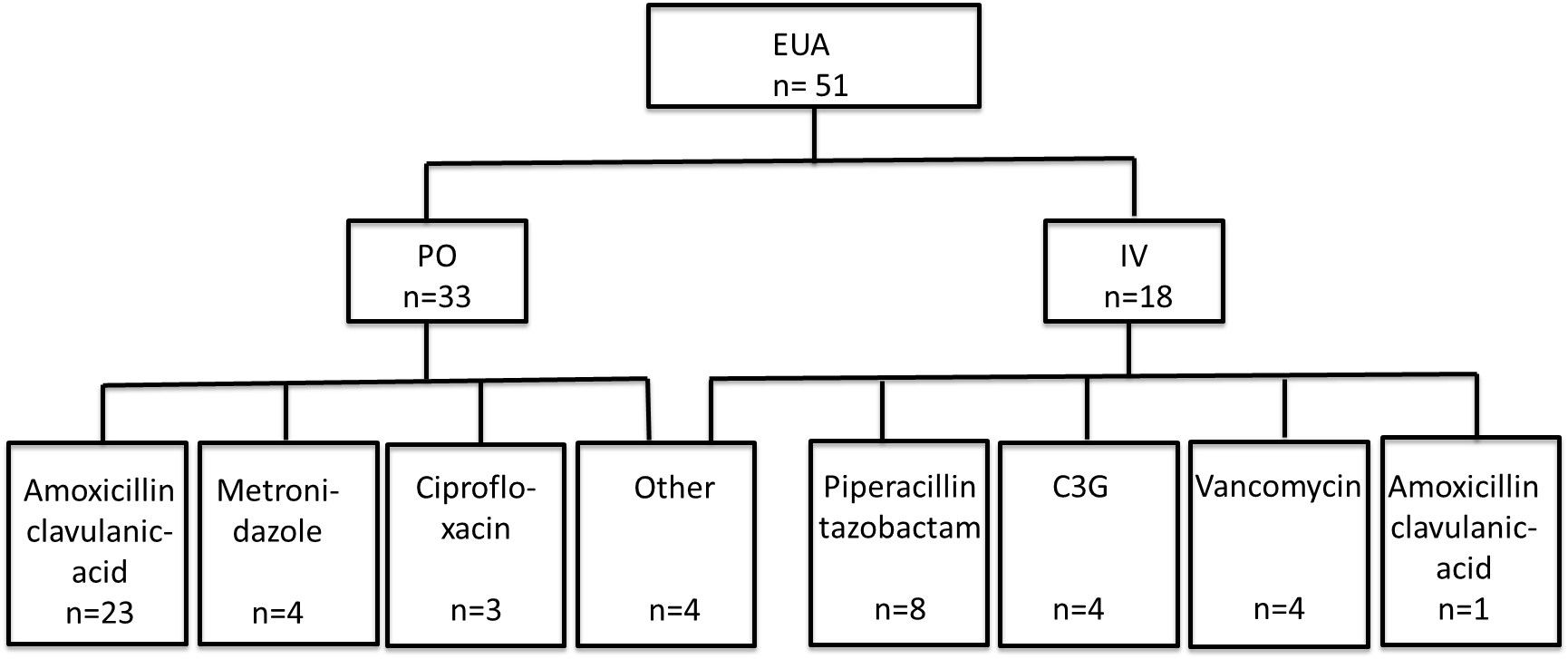
**

**ADDITIONAL FIGURE 2 :** Therapeutic indications of EUA (n=51)

**ADDITIONAL FIGURE 3:** Main bacteria phylum in blood at baseline in the overall population (n=35)

**
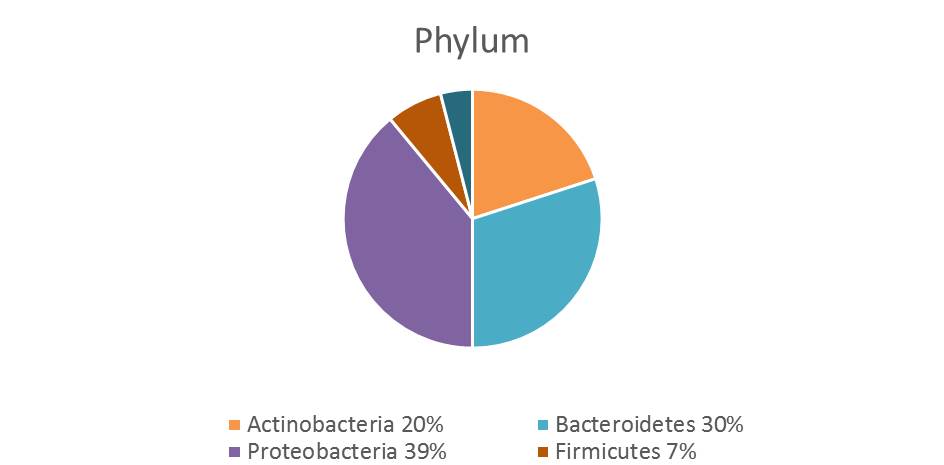
**

**ADDITIONAL FIGURE 4:** LEFSE diagram of blood microbiome at M0 according to tumor response

**ADDITIONAL FIGURE 5:** LEFSE diagram of blood microbiome at M0 according to clinical benefit

**ADDITIONAL FIGURE 6:** LEFSE diagram of blood microbiome at M2 according to early use of antibiotic (EUA)
